# Two Extremely Preterm Infants Discharged with a Home High-Flow Nasal Cannula for Severe Bronchopulmonary Dysplasia

**DOI:** 10.1155/2024/3266928

**Published:** 2024-05-06

**Authors:** Yuichi Kubo, Takuya Tokuhisa, Hiroshi Ohashi

**Affiliations:** ^1^Department of Neonatology, Imakiire General Hospital, Kagoshima, Japan; ^2^Department of Neonatology, Kagoshima City Hospital, Kagoshima, Japan

## Abstract

Home high-flow nasal cannula (HFNC) use in the neonatal field has become prevalent as a noninvasive respiratory support, but its application in home care remains rare. We report two cases in which a home HFNC was effective in managing extremely low-birth-weight infants with severe bronchopulmonary dysplasia (BPD). Case 1 was a male infant born at 22 weeks' gestation weighing 435 g. Case 2 was a female infant born at 23 weeks' gestation weighing 450 g. Both patients had mothers with chronic placental abruption or chorioamnionitis. They transitioned from invasive mechanical ventilation to nasal CPAP (nCPAP) at 45 days (case 1) and 50 days (case 2) old. Subsequently, at 324 days (case 1) and 90 days (case 2) old, they transitioned to a HFNC, demonstrating stable oxygenation and ventilation, but faced difficulty in removal. Considering the drawbacks of prolonged hospitalization, the patients were discharged using a home HFNC at 404 days (case 1) and 391 days (case 2) old. For case 1, the HFNC was set at 4 L/min of room air and 2 L/min of oxygen, whereas for case 2, it was set at 5 L/min of room air and 1 L/min of oxygen. These settings maintained an SpO_2_ above 90% and a pCO_2_ below 60 mmHg. An HFNC offers advantages over nCPAP owing to its lower invasiveness and reduced discomfort for long-term use. However, reports on the use of a home HFNC for BPD are scarce. In recent years, while premature infant mortality has decreased worldwide, the incidence of BPD has risen, necessitating preparedness for prolonged ventilation in preterm infants. Home ventilators represent a strategy to prevent extended hospitalization, and based on our cases, home HFNC for BPD appears safe and effective, making it potentially useful for managing preterm infants requiring prolonged respiratory support in the future.

## 1. Introduction

High-flow nasal cannulas (HFNCs) have become a major respiratory device in the neonatology field. Recently, some cases of home HFNC have been reported [1, 2]. We herein report two cases in which home HFNC was sufficient to manage an extremely preterm infant with severe bronchopulmonary dysplasia (BPD).

## 2. Case Presentation

**Figure 1:**
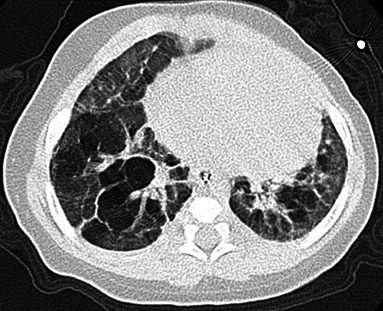
Axial CT on the 222nd DOL of case 1.

**Figure 2:**
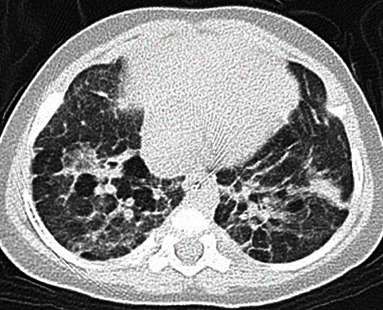
Axial CT on the 244th DOL of case 2.

Both cases 1 and 2 were Japanese neonates without known genetic disorders or anomalies, and both mothers suffered from chronic placental abruption and chorioamnionitis. They were delivered by emergent Caesarian section because of nonreassuring fetal status (case 1) and uncontrollable labor (case 2). Case 1 was born without antenatal corticosteroids, while case 2 was born with them. Both patients developed severe BPD due to severe inflammation associated with chorioamnionitis, followed by bronchial wall thickening and a mixture of partial atelectasis and partial emphysema on computed tomography (CT) (Figures 1 and 2). We used inhaled corticosteroids during intubation and systemic corticosteroids when the respiratory status was alleviated, and diuretics were used during hospitalization and after discharge. Both patients required prolonged intubation, necessitating frequent adjustments of ventilator settings to prevent exacerbation of bronchopulmonary dysplasia (BPD) caused by ventilator-induced lung injury (VILI). Fortunately, neither patient developed serious complications such as tension pneumothorax. Mobility was restricted during intubation, but both patients received rehabilitation after extubation. The detailed clinical courses are described in Table 1.

In both cases, we considered exchanging the HFNC for a low-flow one; however, this notion was abandoned, as case 1 showed respiratory distress under HFNC use, and case 2 showed respiratory distress after withdrawal of the HFNC. We abandoned the restart of nasal CPAP (nCPAP) because the patients were too active in their movements to be attached to the nCPAP machine continuously and did not show upper airway obstruction. In addition, we discussed reintubation or tracheostomy, but ultimately decided not to intensify respiratory support because their respiratory status gradually improved, and their parents rejected this suggestion. At discharge, there were no abnormal neurological signs or symptoms, including intraventricular hemorrhage. At discharge, although their development was delayed by one to two months from the corrected age, they showed good mobility and activity. However, neither patient was able to drink milk well, requiring a nasogastric tube to consume enough milk perhaps because of respiratory distress. We did not perform gastronomy because we hoped that their respiratory status would improve gradually, and their parents did not wish to have the patients undergo surgery. In addition, the patients were diagnosed with pulmonary hypertension caused by BPD, and sildenafil and bosentan were started during hospitalization and continued after discharge. We have obtained the written informed consent from both patients' parents for publication of their cases during their admission.

We chose PrismaVENT50-C® as the home ventilator with an oxygen concentrator. This ventilator is a home ventilator from Löwenstein Medical Technology that can be used for both nCPAP and as an HFNC for neonates, with easy switching between modes, and can deliver from 5 to 60 L/min room air flow with a maximum 15 L/m oxygen flow added from the bypass line. Actually, they used HFNC, not nCPAP; however, we also prepared nCPAP, as home HFNC for BPD has been rare in the neonatology field, and we could not be sure of the safety and efficacy of home HFNC. In both cases, for nCPAP, the PEEP was set to 5 cmH_2_O, and for HFNC, the flow rate was set to 6 L/min. These settings maintained the SpO_2_ at >90% and pCO_2_ at <60 mmHg. We discharged the patients after teaching their parents how to use the device, allowing them to then choose nCPAP or HFNC in accordance with the situation. After discharge, we followed the patients for approximately one year at our clinic, and they used only the HFNC (not nCPAP). Actually, we instructed the parents on the use of mask oxygenation as a response to sudden apnea and desaturation before the patients' discharge. However, no sudden deterioration of the general condition was actually observed at home, and there was no opportunity for resuscitation by the parents. The parents reported that they had no trouble with the home HFNC; they were able to use the device easily, and their children greatly preferred the HFNC over nCPAP. Actually, we provided SpO_2_ monitors for the patients before their discharge and instructed their parents on how to use them. However, after discharge, the infants' development progressed smoothly, and they became too active to wear the monitor easily. Therefore, we told the parents that they could put the monitor on the infants during periods of low activity, such as during sleep, and that they did not need to put the monitor on if the infants' appearance was good while they were actively moving. The patients showed normal growth with regard to weight and height corrected for age and no severe illness, and their respiratory symptoms and pulmonary hypertension gradually improved; therefore, we are trying to switch from the HFNC to a normal nasal cannula. Fortunately, either before or after discharge, neither patient experienced any episodes of sepsis that would have acutely exacerbated BPD or pulmonary hypertension.

## 3. Discussion

HFNCs in the neonatology field are a “new” noninvasive respiratory support that has rapidly become widespread in the last few decades. This device delivers blended oxygen at a high flow rate (usually >1 L/min) [3]. The efficacy of HFNCs has mainly been verified in comparison to nCPAP as temporary respiratory support for respiratory distress after birth or extubation, and it has shown equivalent ability to nCPAP [3, 4]. An HFNC has some benefits over nCPAP, including a reduced invasiveness and simpler interface than that for nCPAP. An HFNC is also reportedly easier to apply, more comfortable, and less likely to cause nasal trauma in neonates than nCPAP [3]. In addition, while pneumothorax is a complication of HFNC introduction [5], its incidence is lower than that with nCPAP. In fact, there was no pneumothorax in our case, as HFNC is less invasive than nCPAP [4].

However, long-term use of an HFNC for chronic respiratory diseases, such as home oxygen therapy, is still not widespread. According to the guidelines on home oxygen therapy for children, home HFNCs have proven successful in managing obstructive sleep apnea and tracheomalacia, but this approach has received little attention [1]. Therefore, there have only been a few clinical reports of home HFNC implementation [1, 2], which is why we included a device with both nCPAP and HFNC settings. To our knowledge, only one study has reported home HFNC use in cases of BPD [6]. However, while that report described several cases of BPD with a home HFNC, salient details, such as the clinical course of the patients, indications for use, and issues with home HFNC use, were lacking. Therefore, the provision of detailed clinical information makes our case valuable.

In recent years, there has been a worldwide reduction in the mortality rate of very preterm neonates, whereas BPD incidence has increased [7]. Thus, we must prepare patients to receive prolonged ventilation, as in our cases. Home ventilation is a countermeasure for preventing prolonged hospitalization, and based on our cases, home HFNC use for BPD seems safe and effective. As mentioned previously, an HFNC is less invasive and more comfortable than nCPAP. These excellent features, which lead to good tolerance, can improve adherence to home ventilator use [2], which is extremely important for ventilator-dependent but active children, such as in our cases.

In conclusion, a home HFNC can be used safely and effectively in infants with severe BPD and is expected to be an essential treatment measure for prolonged ventilation in extremely preterm infants.

## Figures and Tables

**Table 1:** The table of detailed course for two cases.

	Clinical course	The setting of home HFNC
Case 1 Sex: male Gestational age: 22 weeks + 6 days Birth weight: 435 g	Birthday— 45th DOL: invasive ventilation with ETT 45–152nd DOL: nasal CPAP(biphasic mode) (152–170th DOL: invasive ventilation with ETT, 170–193rd DOL: nasal CPAP (biphasic mode), 193–324th DOL: NIV-NAVA because of acute colonic perforation) 324–404th DOL: HFNC 404th DOL: discharge with home HFNC to home At clinic: the parents want to adjust the timing when HFNC was attached or broken off, then we depended on their decision and advised them to elongate the duration of break off	6 L/min (4 L/min of room air and 2 L/min of oxygen) FiO_2_ ≒ 47.3%

Case 2 Sex: Female Gestational age: 23 weeks + 5 days Birth weight: 450 g	Birthday—50th DOL: invasive ventilation with ETT 50–90th DOL: NIV-NAVA 90–391th DOL: HFNC (182–191th DOL: invasive ventilation with ETT, 191–193rd DOL: NIV-NAVA, 193rd—HFNC because of acute exacerbation of PH) 391th DOL: discharge with home HFNC to the rehabilitation hospital At clinic: we first set the timing of break off while the patient is awake, and finally break HFNC off also while she was asleep	6 L/min (5 L/min of room air and 1 L/min of oxygen) FiO_2_ ≒ 34.1%

ETT: endotracheal tube; DOL: day of life; NIV-NAVA: noninvasive neurally adjusted ventilatory assist.

## Data Availability

No data were used to support the findings of this study.
